# Rapid Investigation and Screening of Bioactive Components in Simo Decoction via LC-Q-TOF-MS and UF-HPLC-MD Methods

**DOI:** 10.3390/molecules23071792

**Published:** 2018-07-20

**Authors:** Yingjie He, Pi Cheng, Wei Wang, Sien Yan, Qi Tang, Dongbo Liu, Hongqi Xie

**Affiliations:** 1 Horticulture and Landscape College, Hunan Agricultural University, Changsha 410128, China; yingjiehe272@163.com (Y.H.); picheng55@126.com (P.C.); 18390946378@163.com (W.W.); carryyse@hotmail.com (S.Y.); tangqi@hunau.edu.cn (Q.T.); 2 State Key Laboratory of Subhealth Intervention Technology, Changsha 410128, China; 3 Hunan Co-Innovation Center for Utilization of Botanical Functional Ingredients, Changsha 410128, China; 4 Hunan Provincial Key Laboratory of Crop Germplasm Innovation and Utilization, Hunan Agricultural University, Changsha 410128, China

**Keywords:** quadrupole time-of-flight mass spectrometry, ultrafiltration, HSA ligands, molecular docking, Simo decoction

## Abstract

Simo decoction (SMD), as a traditional medicine, is widely used in the treatment of gastrointestinal dysmotility in China. In this study, a combined method of liquid chromatography quadrupole time-of-flight mass spectrometry (LC-Q-TOF-MS) and ultrafiltration high-performance liquid chromatography molecular docking (UF-HPLC-MD) was efficiently employed to identify and screen bioactive ingredients in SMD. Ninety-four major constituents were identified or tentatively characterized by comparing their retention times and mass spectra with standards or literature data by using LC-Q-TOF-MS, and the ascription of those compounds were classified for the first time. Among them, 13 bioactive ingredients, including norisoboldine, eriocitrin, neoeriocitrin, narirutin, hesperidin, naringin, neohesperidin, hesperitin-7-*O*-glucoside, linderane, poncirin, costunolide, nobiletin, and tangeretin, were primarily identified as the human serum albumin (HSA) ligands at a range of docking scores from −29.7 to −40.6 kJ/mol by UF-HPLC-MD. The results indicate the systematic identification and screening of HSA ligands from Simo decoction guided by LC-Q-TOF-MS and UF-HPLC-MD represents a feasible and efficient method that could be extended for the identification and screening of other bioactive ingredients from natural medicines.

## 1. Introduction

As a traditional Chinese medicine prescription, Simo decoction (SMD) is composed of *Semen arecae*, *Radix linderae*, *Radix aucklandiae*, and *Aurantii fructus*. It has been used abundantly to regulate gastrointestinal function and bloating in clinical applications for a thousand years [1,2]. Literature shows that SMD combined some methods e.g., chewing gum or acupuncture, could enhance bowel function recovery, prevent postoperative ileus, and shorten hospital stay in postoperative patients [3,4]. The positive effects may be due to its participation of the regulation of gastrointestinal hormones of the digestive system, and promotion of gastrointestinal motility by promoting contraction of smooth muscle [5,6]. Despite many clinical treatments having been applied, arecoline, norisobodine, naringin, hesperidin, neohesperidin, and narirutin have been identified as the main effective components [7,8]. Besides, some compounds e.g., narirutin, naringin, hesperidin, neohesperidin, and nobiletin were detected in the plasma of rats [9]. However, chemical compounds of SMD were still not completely identified and systematically classified, and the bioactive ingredients should be further investigated in detail.

The degree to which a drug is protein-bound in plasma has a marked effect on its toxicological, pharmacological, and pharmacokinetic parameters. It is widely believed that only the free concentration, rather than the total drug concentration, can elicit pharmacological responses [10,11]. Human serum albumin (HSA) is the most abundant protein in the circumstance of blood circulation, playing a crucial role of the protein to transport and transmit many endogenous and exogenous constituents such as fatty acids, hormones, and drugs [12,13]. The binding affinity of HSA with drugs is connected to the efficiency of clinical treatment. Therefore, the binding affinity of HSA and drug is an essential parameter that should be carefully analyzed in drug studies [14]. In vitro means have been frequently applied to select HSA ligands from purified extracts of medicinal plants. However, trials based on active compounds need sophisticated and multiple isolation steps which are labor-intensive, time-consuming, and expensive [15]. With the advance of the analytical techniques for active ingredients in complex systems, one method based on ultrafiltration coupled with liquid chromatography mass spectrometry (UF-LC-MS) is considered to investigate the combination between HSA and bioactive compounds [11,16]. Due to its low sample consumption, reuse of receptors (e.g., HSA, enzymes), and obviated need for immobilization, bioactive ingredients have been high-throughput screened and identified via the UF-LC-MS technique [17]. This method enables an efficient separation of the binder–receptor complexes from unbound ingredients [18]. Besides, the binding affinity of the bioactive could be calculated by comparing the ultrafiltration chromatogram and reference chromatogram, by yielding the ratios of the unbound and total amount of single component [11,19]. The structure types could also be obtained by the MS/MS system. In addition, molecular docking has also been employed as a crucial tool to select bioactive components, and has exhibited efficient screening ability from multiple targets with a substantial degree of accuracy, time-saving, and cost-effectiveness in drug discovery [20,21]. It could therefore be an appropriate assistant in the ultrafiltration screening method.

**Figure 1:**
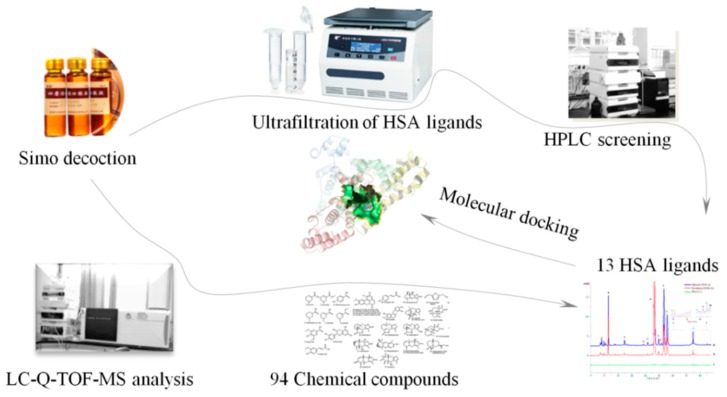
Strategy based on liquid chromatography quadrupole time-of-flight mass spectrometry (LC-Q-TOF-MS) and ultrafiltration high-performance liquid chromatography molecular docking (UF-HPLC-MD) method to identify and screen the bioactive ingredients in Simo decoction (SMD). HSA = human serum albumin; HPLC = high-performance liquid chromatography.

Inspired by the applications mentioned above, a simplified and efficient strategy on the strength of liquid chromatography quadrupole time-of-flight mass spectrometry (LC-Q-TOF-MS) and ultrafiltration high-performance liquid chromatography molecular docking (UF-HPLC-MD) to investigate the bioactive ingredients in SMD was developed, as depicted in Figure 1. To the best of our knowledge, this is the first time that LC-Q-TOF-MS and UF-HPLC-MD have been integrated in the identification and screening of major bioactive components from SMD. The LC-Q-TOF-MS technique could improve the fast detection of chemical compounds, while UF-HPLC-MD supports an approach for the recognition of bioactive ligands of HSA, predicting their binding sites and illustrating more information about the interaction mechanisms between receptor and active ligands [22]. The present study illustrates and explains the practical application of the bioactive compounds of SMD for the clinical treatment of gastrointestinal diseases.

## 2. Materials and Methods

### 2.1. Chemicals and Reagents

SMD, used for gastrointestinal dysmotility in China (approval number: guo-yao-zhun-zi Z20025044; specification: 10 mL/division), was obtained from Hunan Hansen Pharmaceutical Company, Ltd. (Yi Yang, China). HSA was acquired from Sigma Chemical Co. (St. Louis, MO, USA), standards, including arecaidine, arecoline, norisoboldine, linderane, costunolide, dehydrocostus lactone, synephrine, rutin, limonin, eriocitrin, narirutin, naringin, hesperidin, heohesperidin, poncirin, naringenin, hesperetin, nobiletin, tangeretin, and sorbic acid with a purity of over 98%, were purchased from Yuan-ye Bio-Technology Co., Ltd. (Shanghai, China). The formic acid, acetonitrile, and methanol used for HPLC analysis were chromatographic grade and purchased from Sinopharm Chemical Reagent Co., Ltd. (Shanghai, China).

### 2.2. HPLC Conditions

An Agilent 1260 HPLC system (Agilent Technologies, Palo Alto, CA, USA), equipped with a quat pump, an automatic sampler with a 20 μL sample loop, a thermostat of column, a diode array detector (DAD), and an Agilent ChemStation (Agilent Technologies, Palo Alto, CA, USA) had been employed to analyze samples. A Waters-XTerra™ C18 column (250 mm × 4.6 mm, 5 μm, Waters Corp., Milford, MA, USA) was performed for the chromatographic separation of SMD.

### 2.3. Q-TOF-MS Apparatus

Identification of mass spectrum was employed on an accurate mass spectrometer of Agilent 6530 Q-TOF-MS (Agilent Technologies, Palo Alto, CA, USA). Chromatographic separation was employed on an Agilent-ZORBAX SB-C18 column (250 mm × 4.6 mm, 5 μm, Agilent Technologies, Palo Alto, CA, USA), and the effluent of the HPLC mobile phase was split and guided into the electrospray ionization (ESI) source. Parameter conditions were performed as following: capillary voltage, 3500 V; nebulizer pressure, 50 psi; nozzle voltage, 1000 V; flow rate of drying gas, 6 L/min; temperature of sheath gas, 350 °C; flow rate of sheath gas, 11 L/min; skimmer voltage, 65 V; OCT1 RF Vpp, 750 V; fragmentor voltage, 135 V. The spectra data were recorded in the range of *m*/*z* 100–1000 Da in a centroid pattern of full-scan MS analysis mode. The MS/MS data of the selected compounds were obtained by regulating diverse collision energy (18–45 eV).

### 2.4. Sample Preparations

The SMD for ultrafiltration and LC-MS were filtered through a 0.22 μm membrane, then diluted to 1:10 *V*/*V* with a buffer solution of ammonium acetate buffer solution (ABS; 10 mM, pH 7.4) before experiments. The HSA (600 µM) was dissolved in ABS and prepared as the work solution

### 2.5. UF-HPLC-Based Binding Assay

The procedure of screening was manipulated according to the approach of previous research and consisted of three steps: incubation, washing, and dissociation [11,23]. Briefly, 100 µL of tested SMD solution was incubated with 200 µL HSA (600 µM) and 200 µL buffer solution for 20 min at 37 °C. Meanwhile, denatured HSA solution (boiled for 15 min in a water bath) was used as the negative control in the same manner. The incubated solutions were then filtered through ultrafiltration devices (Millipore Corp., Billerica, MA, USA) with a 30 kDa molecule weight cut-off membrane (Millipore AmiconUltra-0.5 mL, item: UFC503096) and centrifuged at 14,000× *g* to separate the non-specific ingredients from the HSA-ligand complexes for 15 min at room temperature. The residues were then washed with 200 µL of buffer solution by centrifugation to remove the unbound components three times. The ligands showing specific binding to HSA were then released from the mixtures by elution with 400 µL 50% methanol (pH = 3) for 20 min, and then centrifuged at 14,000× *g* for 15 min at room temperature, a process which was repeated twice. The dissociated filtrates were combined and added to 1000 µL by 50% methanol and then directly analyzed.

### 2.6. Molecular Docking Study

To further study the coactions of the bioactive ligands with HSA, a molecular docking study which could conjecture the interactions of ligands within the constraint of receptors binding sites was performed in silico. In the prediction, the initial three-dimensional structure of the HSA was acquired from the Protein Data Bank (PDB, ID: 1E7I). The binders and water molecules were removed from the crystal structure of HSA by using PyMOL (Schrödinger LLC, New York, NY, USA) [11]. The 3D structures of the ligands were drawn and converted using ChemBioDraw Ultra and ChemBio 3D Ultra (Cambridgesoft Corp., Waltham, MA, USA) [11].

The AutoDock Vina [24] was employed for the docking simulation of these ligands. The docking steps were performed according to the protocol described by Ma et al. [25], with some modifications. Due to the various molecule sizes, in the first round of docking, each grid computation was calculated covering all amino acid residues of HSA to recognize the binding sites, and the simulation was then performed with flexible docking of all molecules in HSA. The grid was then concentrated on the center of Sudlow’s site I (60 Å × 60 Å × 60 Å, 0.375 Å, central coordinates *x* = 30.938, *y* = 13.241, and *z* = 7.960) and Sudlow’s site II (60 Å × 60 Å × 60 Å, 0.375 Å, central coordinates *x* = 9.491, *y* = 5.575, and *z* = 18.576), respectively, to find the appropriate binding sites [11]. The calculation of docking score was repeated three times for each ligand. Finally, PyMOL was used to present the docking results.

## 3. Results and Discussion

### 3.1. Optimization of HPLC Conditions

Because of the complicated compositions of four main traditional Chinese medicines, the adequate separation of the aimed constituents is a challenging and essential procedure for HPLC analysis [23]. The SMD was rich in flavonoids, alkaloids, and lactone compounds, and therefore, in the HPLC analytical procedures, the separation conditions containing the mobile phase system, column detection wavelength (nearly higher absorption), temperature, and so on should be investigated. Acid is known to improve separation for constituents with hydroxyl groups by reducing the tailing of the chromatographic peaks. Therefore, formic acid was added to the mobile phase composed of solvents A (0.1% formic acid in water) and B (acetonitrile) [26], and a flow rate of gradient elution was elected at 0.7 mL/min. In consideration of the variety of constituents in SMD in previous pre-experiments, the solvent gradient of the mobile phase was finally optimized as follows: 15% B for 0–5 min, 15–20% B for 5–15 min, 20–25% B for 15–30 min, 25–65% B for 30–42 min, 65–90% B for 42–45 min. The programmed wavelength was selected at 284 nm via comparison of the higher absorption of the main compounds. The column temperature was maintained at 30 °C and the volume of injection was 5 µL.

### 3.2. Identification of Constituents in SMD

**Figure 2:**
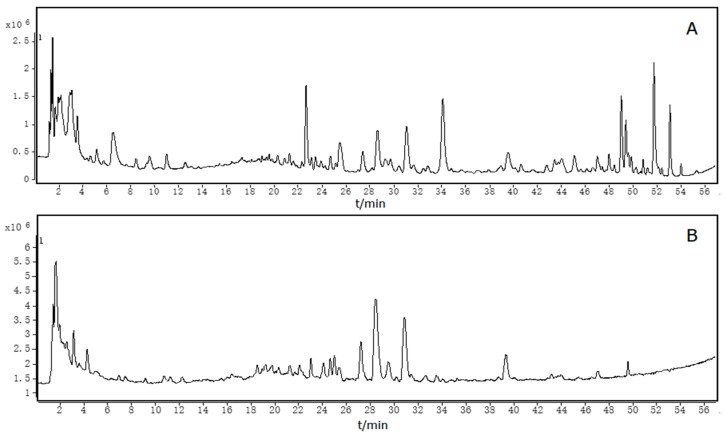
Total ion chromatography of Simo decoction (SMD) in positive (**A**) and negative (**B**) modes.

**Figure 3:**
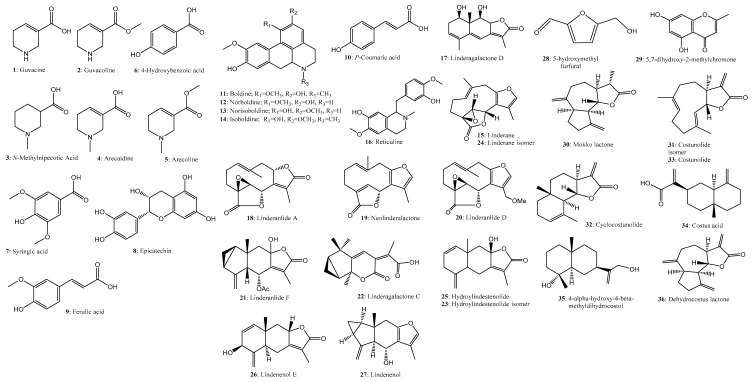

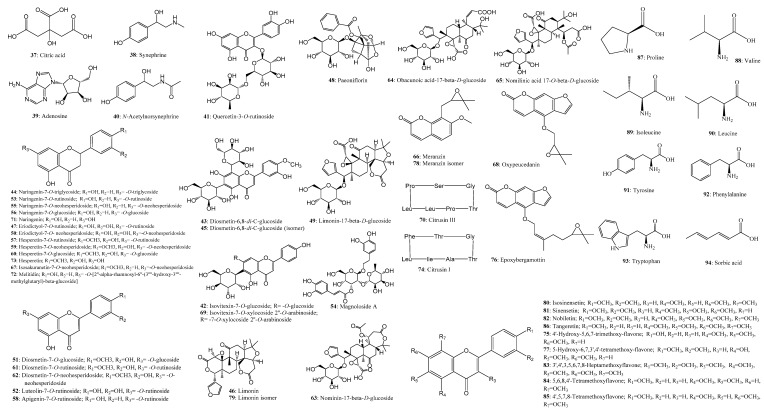
The compounds were identified or preliminarily assigned from Simo decoction (SMD) based on the time-of flight-mass spectrometer.

As many as 94 compounds were identified as the main constituents by ESI-Q-TOF-MS in the positive and negative ion mode (Figure 2), and their origin was classified according to the chemical information of single herb and literatures (Table 1). Compounds **4**, **5**, **13**, **15**, **33**, **36**, **38**, **41**, **46**, **47**, **53**, **55**, **57**, **59**, **67**, **71**, **73**, **82**, and **86** were unambiguously identified as arecaidine [27,28], arecoline [27,28], norisoboldine [29], linderane [30,31,32], costunolide [33,34], dehydrocostus lactone [33,34], synephrine [35], rutin [36], limonin [37,38,39], eriocitrin [36,40], narirutin [36,41], naringin [36,41], hesperidin [36,41], neohesperidin [36,41], poncirin [36,41], naringenin [36], hesperetin [36], nobiletin [36,42], and tangeretin [36,42], respectively, by comparison of the retention time, absorption wavelengths, and *m*/*z* values with the standards and values reported in the literature. The remaining compounds could be tentatively assigned by comparing the fragmentation patterns, the accurate mass data (absolute value of error < 5 ppm), and the formula predictor software (Table 1). The chemical structures of these compounds were drawn clearly as shown in Figure 3.

For example, compound **12** had [M + H]^+^ ions *m*/*z* at 314.1372 yielding the product ions *m*/*z* at 297.1125 [M + H-17]^+^, 265.0839 [M + H-17-32]^+^, and 237.0743 [M + H-17-32-28]^+^, and compound **14** generated [M + H]^+^ ions *m*/*z* at 328.1534 yielding the similar product ions *m*/*z* at 297.1110 [M + H-31]^+^, 265.0859 [M + H-31-32]^+^, and 237.0627 [M + H-31-32-28]^+^, both of which had coincident ions with compound **13** and were tentatively assigned as norboldine [29,31] and isoboldine [29,31], respectively. Similarly, compound **16** was initiatively detected as reticuline according to the ions at 330.1691 [M + H]^+^, 299.1472 [M + H-31]^+^, and 192.0682 [M + H-138]^+^ [29,31].

The sugar parts in *O*-glycosylflavone, such as neohesperidose (1→2) and rutinose (1→6) could be distinguished because neohesperidose in glycosides could yield a stronger abundance of parent nuclei contrasted with rutinose-contained glycosides, and thus could be identified by their characteristic fragmentation behaviors [36]. For example, ion *m*/*z* at 273 of naringin was higher than naritutin, and ion *m*/*z* at 303 of neohesperidin was higher than hesperidin in ESI^+^ mode. Compounds **47** and **50** had the same [M + H]^+^ ions at *m*/*z* 597 and molecular formula of C_27_H_32_O_15_. Compound **50** presented product ions *m*/*z* at 451 [M + H-146]^+^ and 289 [M + H-146-162]^+^, exhibited the same ions with compound **47**, and could yield a much higher abundance of fragment ions at *m*/*z* 289 when compared with that of compound **47** (eriocitrin), suggesting that it contained neohesperidose (1→2), and it was accurately identified as neoeriocitrin. Compound **60** showed [M + H]^+^ ion at *m*/*z* 465.1387 and [M − H]^−^ ion at *m*/*z* 463.1252, and produced the parent ion at *m*/*z* 303.0861 [M + H]^+^, with this compound being preliminarily identified as hesperitin-7-*O*-glucoside. Compound **44** of *m*/*z* 741.2245 [M − H]^−^ produced product ions at 579.1833 [M − H-162]^–^, 417.1323 [M − H-2 × 162]^–^, and 271.0756 [M − H-3 × 162]^–^ that was identified as naringenin-7-*O*-triglycoside [36,40]. The remaining ingredients were similarly analyzed and classified by referring to the original medical plants of SMD as shown in Table 1.

### 3.3. Optimization of Screening Conditions

Working factors of pH and temperature influenced the activity of HSA, time of incubation influenced the binding degree of binders, eluting steps removed the disturbance of unbound compounds, and dissolution reagent was necessary for the dissociation of HSA-drug complexes. Optimum conditions in the study were established by referring to related studies [56,57,58] which performed single factor experiments in previous work. This study was performed at 37 °C (physiological temperature) with pH 7.4 (the plasma condition) to provide optimal reaction conditions. HSA concentration (from 100 μM to 800 μM), incubation time (from 0 min to 50 min), eluting time, and dissolution reagent (methanol of diverse concentration and pH) were improved before experiments. The results showed that each bioactive ligand could be obtained with the best binding affinity when the concentration of HSA was 600 μM to avoid competitive binding, and the incubation time was set at 20 min. ABS was employed as an eluting solution in triplicate, and a 50% methanol solution (pH 3) was optimally chosen to dissociate HSA-drug complexes.

### 3.4. Screening Bioactive HSA Ligands from SMD

**Figure 4:**
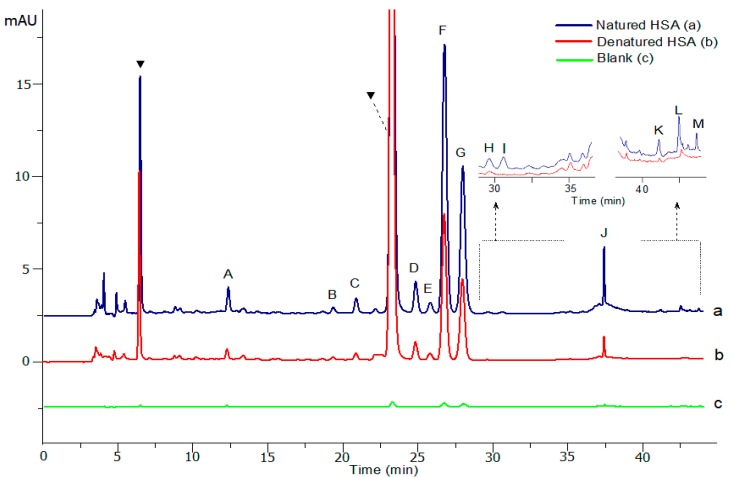
The high-performance liquid chromatography (HPLC) chromatograms for screening of the human serum albumin (HSA) ligands in Simo decoction (SMD) after ultrafiltration and dissociation procedures. The blue solid line represents HPLC profile of SMD sample mixed with natured HSA, and the red and green lines represent HPLC profiles of SMD sample mixed with denatured HSA and buffer solution, respectively. Bioactive ligands (A–M) were identified as norisoboldine (A), eriocitrin (B), neoeriocitrin (C), narirutin (D), hesperidin (E), narigin (F), neohesperidin (G), hesperidin-7-*O*-glucoside (H), linderane (I), neoponcirin (J), costunolide (K), nobiletin (L), and tangeretin (M), respectively. Triangle (▼) represents a compound with a high response but no specific binding named “frequent hitters”.

Using the above screening method, 15 binders were identified as “tight-binding” ligands (Figure 4). However, not all the binders bound to the HSA are specific ligands because some are just ‘‘frequent hitters’’, unselectively clogging the protein by hydrophobic interaction without any specific interactions [59], even though the washing procedures were performed. To distinguish between specific ligands and ‘‘frequent hitters’’, the method of ultrafiltration and dissociation could be efficiently combined. In short, if one compound in the complicated sample is able to interact with a specific target receptor, the peak area of the bound constituent will significantly increase in the total ion chromatogram after dissociation from the drug-protein complexes. In this way, the UF-HPLC assay could rapidly screen and identify the ligand-receptor complexes from unbound or nonspecific binding compounds, by directly comparing the chromatogram peak areas between natured and denatured HSA after ultrafiltration, as shown in (a) and (b) in Figure 4.

Based on the variation of the chromatographic proportion before and after incubation with natured and denatured HSA, the real reduced peak areas can be used to determine the degree of affinity between the ligand and the enzyme. The binding degree (*BD*) was calculated as follows:

(1) $$BD=\frac{An-Ad}{As}\times 100\%$$

where *As*, *An*, and *Ad* represent the peak areas obtained from the SMD sample, natured and denatured HSA after dissociation, respectively. The results in Table 2 suggest that flavonoids and alkaloids could exert good affinity activity to HSA. Finally, 13 bioactive ingredients (A–M), containing norisoboldine, eriocitrin, neoeriocitrin, narirutin, hesperidin, naringin, neohesperidin, hesperitin-7-*O*-gulcoside, linderane, poncirin, costunolide, nobiletin, and tangeretin, were preliminarily identified as the specific HSA ligands.

### 3.5. Repeatability of Ultrafiltration

Due to the potential for nonspecific binders to the HSA to lead to erroneous calculation, the repeatability of the bioactive ingredients in the SMD during ultrafiltration was studied. The repeatability showed the degree of affinity of these specific ingredients in SMD exhibiting large variety from 9.8–26.1% as shown in Table 2, which might be caused by their structure types and proportions. Besides, the bioactivities of the targets might be affected by not only the binding properties but also the drug-like properties [56], and the complicated components exhibited competitive relationships. Therefore, the binding degree in complex compounds might be different from that of single compounds. The relative standard deviation (RSD) of binding degrees of these binders was below 12.7%, indicating that each of the bioactive ligands could interact well with HSA.

### 3.6. Analysis of Molecular Docking

In order to expound how bioactive drugs conjugate with HSA, a molecular docking simulation was used for further illustration in the active sites and binding degree of the ligands on HSA. The interactions of drugs with HSA typically occur at two major hydrophobic sites, known as Sudlow’s site I (subdomain IIA) and site II (subdomain IIIA), which are located in subdomains [57]. Many studies have verified that warfarin and ibuprofen were specific binders for site I and site II, respectively. In this study, the mode of specific docking (grid in site I and site II) showed more specific affinity than the full grid mode, indicating that these components were appropriate ligands of HSA. The simulation scores of the drugs to the two binding sites are listed in Table 2.

**Figure 5:**
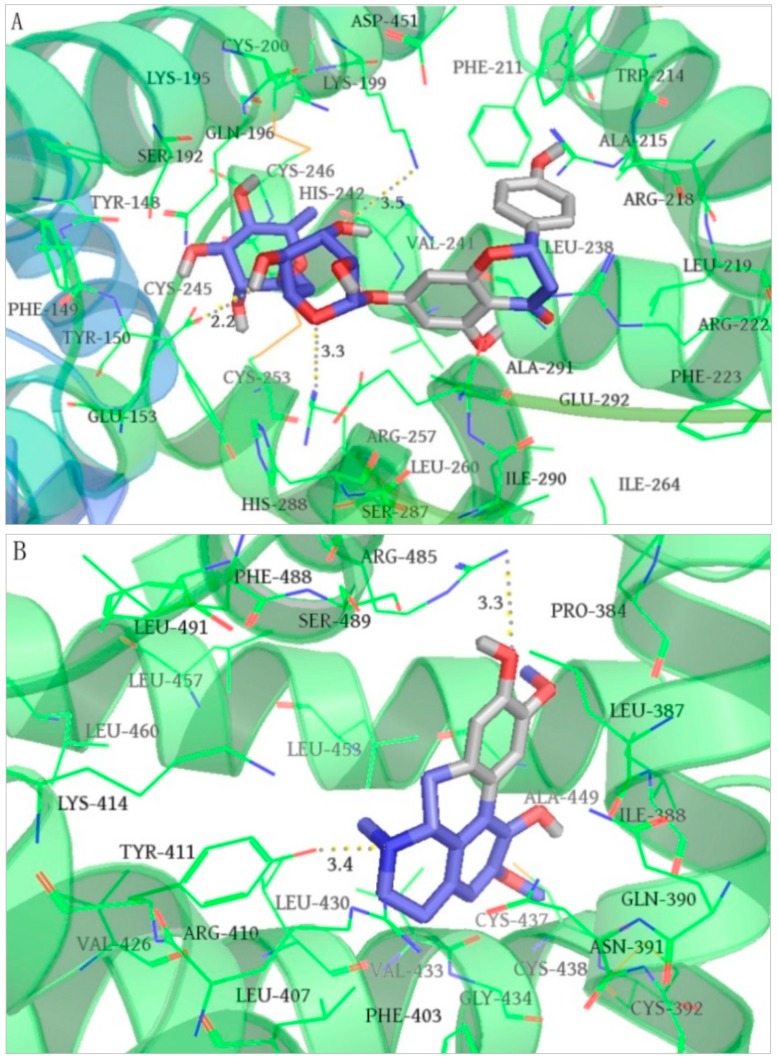
Molecular docking of narirutin docked to site I (**A**) and norisoboldine docked to site II (**B**) of human serum albumin (HSA), respectively (Ligands were shown in stick form and gray dashed lines were hydrogen bonds. The figure was prepared with PyMol. The interactions between bioactive ligands and binding sites were detailed in the article).

To further investigate the interactions between ligands and binding sites, the ligands, narirutin and norisoboldine, with the highest scores of binding site I and site II, respectively, were analyzed. As shown in Figure 5A, narirutin easily inserted into site I with a docking score of −40.6 kJ/mol and was mainly surrounded by 30 amino acid residues within a range of 4 Å. These residues are believed to be important in the binding affinity. Three hydrogen bonds (dash lines) were formed on the 7-rutinose with Glu153, Lys199, and Arg257, respectively. Hydrophobic bonds were generated on the mother nucleus of the flavanone surrounded with 14 amino acid residues, as follows: Phe211, Trp214, Ala215, Arg218, Leu219, Arg222, Phe223, Leu238, Val241, Arg257, Leu260, Ile264, Ile290, and Ala291. As shown in Figure 5B, norisoboldine efficiently bound with site II and was mainly surrounded by 25 amino acid residues within a range of 4 Å. Two hydrogen bonds were formed on the 6-*N* and 9-C (OH) with Tyr411 and Arg458, respectively. Pro384, Leu387, Ile388, Phe403, Leu407, Val426, Leu430, Val 433, Ala449, Leu453, Leu457, Leu460, Phe488, and Leu491 surrounded the molecule forming hydrophobic bonds with a docking score at −36.1 kJ//mol.

It was found that the interactions between HSA and the flavonoids were dependent on the structures of the flavonoids. The glycoside of flavonoid was very important for the affinity degree, which mainly formed hydrogen bonds, while the A, B, and C rings supported hydrophobic bonds. In addition, in site I, as the number of methoxyl groups decreased, the affinity degree might increase. In site II, the docking scores of polymethoxy flavonoids were higher than at site I. This phenomenon was consistent with the tendency of site I to bind bulky heterocyclic anionic compounds and site II to aromatic carboxylates [57]. Moreover, the effect of a hydroxyl on glycosyl was found to be less than that of a hydroxyl on the parent nucleus, perhaps because of the large area of steric hindrance [58]. Although the current simulation studies could be considered efficient and reasonable, we also expect to further apply more advanced methods, such as fluorescence or X-ray, to explain the mechanisms of interactions between the bioactive ingredients and the related receptors, as well as the establishment of animal models to illustrate the metabolic pathways of effective constituents to clarify the pharmacological effects of SMD in future research.

## 4. Conclusions

In the current study, we established a simplified and effective strategy based on LC-Q-TOF-MS and UF-HPLC-MD for the identification of complicated ingredients and the screening of bioactive HSA ligands from SMD. A total of 94 compounds were identified or tentatively speculated by LC-Q-TOF-MS. Among them, nine compounds were derived from *Semen arecae*, 28 compounds were derived from *Radix linderae*, nine compounds were derived from *Radix aucklandiae*, and 40 compounds were derived from *Aurantii fructus*, in addition to the speculation of a further eight common compounds (e.g., amino acids). Flavonoids were abundant in these identified compounds in SMD (Table 1). In addition, HSA binders from SMD were screened by the established UF-HPLC-MD method. A total of 13 bioactive ingredients was primarily illustrated as the specific HSA ligands in SMD which may be the main medicinal components. Molecular docking was employed for further illustration in the active site and binding degree of bioactive ligands on HSA.

SMD is widely used in the clinical treatment of gastrointestinal dynamic disorder, and these results provide reliable data to support the pharmacological research of SMD in the future. They also provide a reference for the reasonable combination of SMD with other methods or drugs in the treatment of gastrointestinal dysmotility. In addition, and compared with the conventional bioassay approach, the proposed strategy enables the rapid illustration of the identification and screening of bioactive components from complex mixtures.

## Figures and Tables

**Table 1:** Identification of constituents from Simo decoction (SMD) by liquid chromatography quadrupole time-of-flight mass spectrometry (LC-Q-TOF-MS) analysis in positive and negative ion modes.

No.	T_R_ (min)	ESI+ (*m*/*z*)	ESI− (*m*/*z*)	Fragment Ions (Positive/Negative)	MW (Mea.)	MW (MFG)	Formula	Compound	Ref.	Error (ppm) ^b^
***Areca catechu***										
**01**	1.899	128.0704		109.0289	127.0631	127.0633	C_6_H_9_NO_2_	Guvacine	[27]	1.61
**02**	2.113	142.0860		/	141.0787	141.0790	C_7_H_11_NO_2_	Guvacoline	[27]	1.96
**03**	3.001	144.1021		/	143.0948	143.0946	C_7_H_13_NO_2_	*N*-Methylnipecotic Acid	[28]	−1.06
**04**	5.163	142.0864		124.0252, 109.0289	141.0791	141.0790	C_7_H_11_NO_2_	Arecaidine ^a^	[27,28]	−0.80
**05**	7.021	156.1018		127.0410	155.0946	155.0946	C_8_H_13_NO_2_	Arecoline ^a^	[27,28]	0.24
**06**	16.247		137.0240		138.0312	138.0317	C_7_H_6_O_3_	4-Hydroxybenzoic acid	[43]	3.23
**07**	17.508	199.0597			198.0525	198.0528	C_9_H_10_O_5_	Syringic acid	[44]	1.62
**08**	19.379	291.0866			290.0793	290.0790	C_15_H_14_O_6_	Epicatechin	[44]	−0.93
**09**	25.337		193.0500		194.0572	194.0579	C_10_H_10_O_4_	Ferulic acid	[43]	3.4
***Radix linderae***										
**10**	5.722	165.0545			164.0472	164.0473	C_9_H_8_O_3_	*p*-Coumaric acid	[29]	0.6
**11**	20.782	328.1546			327.1474	327.1471	C_19_H_21_NO_4_	Boldine	[29,31]	−0.91
**12**	21.558	314.1388		297.1125, 265.0839, 237.0743	313.1315	313.1314	C_18_H_19_NO_4_	Norboldine	[29,31]	−0.26
**13**	22.677	314.1387		297.1141, 265.0787, 237.0619	313.1314	313.1314	C_18_H_19_NO_4_	Norisoboldine ^a^	[29]	−0.12
**14**	23.466	328.1543		297.1110, 265.0859, 237.0627	327.1470	327.1471	C_19_H_21_NO_4_	Isoboldine	/	0.03
**15**	24.093	261.1116		243.1018, 173.0132	260.1043	260.1049	C_15_H_16_O_4_	Linderane ^a^	[30,31,32]	2.07
**16**	24.698	330.1698		330.1691, 299.1472, 192.0682	329.1625	329.1627	C_19_H_23_NO_4_	Reticuline	[29,31]	0.57
**17**	25.234	263.1270			262.1197	262.1205	C_15_H_18_O_4_	Linderagalactone D	[30,31]	3.13
**18**	35.746	277.1068			276.0995	276.0998	C_15_H_16_O_5_	Linderanlide A	[32]	1.01
**19**	40.231	245.1169			244.1096	244.1099	C_15_H_16_O_3_	Neolinderalactone	[30,31]	1.50
**20**	40.590	291.1223			290.1150	290.1154	C_16_H_18_O_5_	Linderanlide D	[32]	1.51
**21**	44.323	305.1376			304.1303	304.1311	C_17_H_20_O_5_	Linderanlide F	[32]	2.54
**22**	46.623	263.1279			262.1207	262.1205	C_15_H_18_O_4_	Linderagalactone C	[30]	−0.57
**23**	47.462	247.1326			246.1254	246.1256	C_15_H_18_O_3_	Hydroylindestenolide isomer	[29]	0.92
**24**	49.032	261.1117			260.1044	260.1049	C_15_H_16_O_4_	Linderane isomer	/	1.64
**25**	50.118	247.1325			246.1252	246.1256	C_15_H_18_O_3_	Hydroylindestenolide	[30,31]	1.53
**26**	50.126	247.1329			246.1256	246.1256	C_15_H_18_O_3_	Lindenenol E	[29]	0.08
**27**	54.009	231.1380			230.1307	230.1307	C_15_H_18_O_2_	Lindenenol	[31]	−0.01
***Radix aucklandiae***										
**28**	11.014	127.0388	/		126.0315	126.0317	C_6_H_6_O_3_	5-HydroxymethylFurfual	[45]	1.24
**29**	25.283	193.0490			192.0417	192.0423	C_10_H_8_O_4_	5,7-dihydroxy-2-methylchromone	[45]	2.89
**30**	44.022	233.1534			232.1461	232.1463	C_15_H_20_O_2_	Mokko lactone	[46]	0.91
**31**	46.101	233.1532			232.1459	232.1463	C_15_H_20_O_2_	Costunolide isomer	/	1.84
**32**	47.095	233.1536			232.1464	232.1463	C_15_H_20_O_2_	Cyclocostunolide	[46]	−0.16
**33**	49.408	233.1530		187.1475, 121.0516	232.1457	232.1463	C_15_H_20_O_2_	Costunolide ^a^	[33,34]	2.75
**34**	49.895	235.1691			234.1618	234.1620	C_15_H_22_O_2_	Costus acid	[46]	0.71
**35**	51.221	239.2003			238.1930	238.1933	C_15_H_26_O_2_	4-*α*-hydroxy-4-*β*-methyldihydrocostol	[46]	1.2
**36**	54.001	231.1373			230.1301	230.1307	C_15_H_18_O_2_	Dehydrocostus lactone ^a^	[33,34]	2.69
***Aurantii fructus***										
**37**	3.201		191.0189		192.0262	192.0270	C_6_H_8_O_7_	Citric acid	[37,47]	4.06
**38**	3.802	168.1017			167.0944	167.0946	C_9_H_13_NO_2_	Synephrine ^a^	[35]	1.45
**39**	9.369	268.1035			267.0962	267.0968	C_10_H_13_N_5_O_4_	Adenosine	[37]	2.03
**40**	17.108	196.0967			195.0894	195.0895	C_10_H_13_NO_3_	*N*-Acetylnorsynephrine	/	0.8
**41**	20.439	611.1590		465.0874, 303.0511	610.1518	610.1534	C_27_H_30_O_16_	Quercetin-3-*O*-rutinoside (Rutin) ^a^	[36]	2.53
**42**	21.337	595.1659	593.1500		594.1587	594.1585	C_27_H_30_O_15_	Isovitexin-7-*O*-glucoside (Saponarin)	[48]	−0.31
**43**	21.903	625.1766	623.1615	301.0723	624.1693	624.1690	C_28_H_32_O_16_	Diosmetin-6,8-*di*-*C*-glucoside	[47]	−0.46
**44**	22.234		741.2245	579.1833, 417.1323, 271.0756	742.2318	742.2320	C_33_H_42_O_19_	Naringenin-7-*O*-triglycoside	[36,40]	0.36
**45**	22.377	625.1761			624.1688	624.1690	C_28_H_32_O_16_	Diosmetin 6,8-*di*-*C*-glucoside (isomer)	[47]	0.41
**46**	23.947	471.2007			470.1935	470.1941	C_26_H_30_O_8_	Limonin ^a^	[37,38,39]	1.28
**47**	24.235	597.1813	595.1663	435.1278, 417.1185, 331.1826, 289.0702	596.1740	596.1741	C_27_H_32_O_15_	Eriodictyol-7-*O*-rutinoside (Eriocitrin) ^a^	[36]	0.12
**48**	24.460	481.1683			480.1610	480.1632	C_23_H_28_O_11_	Paeoniflorin, Albiflorin	[40]	4.44
**49**	24.603		649.2501		650.2573	650.2575	C_32_H_42_O_14_	Limonin-17-*β*-d-glucoside	[39,49]	0.16
**50**	25.124	597.1807	595.1656	451.1287, 289.0699	596.1735	596.1741	C_27_H_32_O_15_	Eriodictyol-7-*O*-neohesperidoside (Neoeriocitrin)	[36,37]	1.06
**51**	26.946		461.1067		462.1140	462.1162	C_22_H_22_O_11_	Diosmetin-7-*O*-glucoside	[35]	4.85
**52**	26.986	595.1653		463.1303, 287.0559	594.1579	594.1585	C_27_H_30_O_15_	Luteolin-7-*O*-rutinoside (Veronicastroside)	/	0.9
**53**	27.421	581.1853	579.1705	435.1274, 273.0757	580.1781	580.1792	C_27_H_32_O_14_	Naringenin-7-*O*-rutinoside (Narirutin) ^a^	[36,41]	1.97
**54**	27.989	625.2107		643.1461, 267.1224	624.2034	624.2054	C_29_H_36_O_15_	Magnoloside A	[37,47]	3.31
**55**	28.694	581.1857	579.1687	435.1278, 419.1330, 273.0754, 153.0186	580.1785	580.1792	C_27_H_32_O_14_	naringenin-7-*O*-neohesperidoside (Naringin) ^a^	[36,41]	1.26
**56**	29.032	435.1274		273.0757	434.1201	434.1213	C_21_H_20_O_10_	Naringenin-7-*O*-glucoside		2.71
**57**	29.692	611.1965	609.1803	465.1432, 303.0858, 273.0757	610.1891	610.1898	C_28_H_34_O_15_	Hesperetin-7-*O*-rutinoside (Hesperidin) ^a^	[36,41]	1.13
**58**	30.385	579.1708	577.1549	433.1323, 271.0596	578.1636	578.1636	C_27_H_30_O_14_	Apigenin-7-*O*-rutinoside (Isorhoifolin)	[40]	−0.01
**59**	31.051	611.1962	609.1811	465.1434, 303.0862, 153.0188	610.1889	610.1898	C_28_H_34_O_15_	Hesperetin-7-*O*-neohesperidoside (Neohesperidin) ^a^	[36,41]	1.37
**60**	31.121	465.1395		331.1881, 303.0861, 155.0372, 121.0216	464.1322	464.1319	C_22_H_24_O_11_	Hesperitin-7-*O*-glucoside	[37]	−0.73
**61**	31.638	609.1819		463.1409, 301.0723	608.1747	608.1741	C_28_H_32_O_15_	Diosmetin-7-*O*-rutinoside (Diosmin)		−0.88
**62**	32.515	609.1806		463.1411, 301.0723	608.1734	608.1741	C_28_H_32_O_15_	Diosmetin-7-*O*-neohesperidoside (Neodiosmin)	[47]	1.12
**63**	32.531		693.2756		694.2829	694.2837	C_34_H_46_O_15_	Nominin-17-*β*-d-glucoside	[39,49]	1.12
**64**	32.787		651.1541		652.1614	652.1639	C_29_H_32_O_17_	Obacunoic acid-17-*β*-d-glucoside	[39]	3.92
**65**	33.509		711.2850		712.2923	712.2942	C_34_H_48_O_16_	Nomilinic acid 17-*O*-*β*-d-glucoside	[39,49]	2.74
**66**	34.361	261.1120			260.1047	260.1049	C_15_H_16_O_4_	Meranzin hydrate	[50]	0.67
**67**	39.559	595.2016	593.1875	449.1505, 287.0917	594.1944	594.1949	C_28_H_34_O_14_	Isosakuranetin-7-*O*-neohesperidoside, (Poncirin) ^a^	[36,41]	0.77
**68**	39.546	287.0913			286.0840	286.0841	C_16_H_14_O_5_	Oxypeucedanin	[48]	0.48
**69**	41.776	697.1975			696.1901	696.1902	C_31_H_36_O_18_	Isovitexin-7-*O*-xylocoside 2″-*O*-arabinoside	[40]	0.05
**70**	42.803	728.3970			727.3896	727.3905	C_36_H_53_N_7_O_9_	Citrusin III	[35,51,52]	1.15
**71**	43.149		271.0609		272.0682	272.0685	C_15_H_12_O_5_	Naringenin ^a^	[36]	1.18
**72**	44.264	725.2283			724.2210	724.2215	C_33_H_40_O_18_	Melitidin	[53]	0.65
**73**	45.375		301.0714		302.0787	302.0790	C_16_H_14_O_6_	Hesperetin ^a^	[36]	1.27
**74**	47.003	704.3968			703.3895	703.3905	C_34_H_53_N_7_O_9_	Citrusin I	[52]	1.40
**75**	47.187	329.1023		314.0762, 299.0543	328.0950	328.0947	C_18_H_16_O_6_	Monohydroxytrimethoxyflavone	[54]	−1.08
**76**	47.976	355.1533			354.1460	354.1467	C_21_H_22_O_5_	Epoxybergamottin or Cnidicin	[55]	2.01
**77**	48.440	359.1119		344.0877, 326.0771	358.1046	358.1053	C_19_H_18_O_7_	5-Hydroxy-6,7,3′,4′-tetramethoxy-flavone	[54]	1.74
**78**	49.028	261.1117			260.1044	260.1049	C_15_H_16_O_4_	Meranzin, IsoMeranzin	[50]	1.64
**79**	49.634	471.2005			470.1932	470.1941	C_26_H_30_O_8_	Limonin isomer	[37,38]	1.76
**80**	50.227	373.1276		358.1024, 343.0811	372.1204	372.1209	C_20_H_20_O_7_	5,7,8,3′,4′-Pentamethoxyflavone (Isosinensetin)	[36,54]	1.44
**81**	50.853	373.1278		358.1036, 343.0812	372.1205	372.1209	C_20_H_20_O_7_	5,6,7,3′,4′-Pentamethoxyflavone (Sinensetin)	[54]	1.04
**82**	51.721	403.1385		388.1025, 373.1253	402.1312	402.1315	C_21_H_22_O_8_	5,6,7,8,3′,4′-Hexamethoxyflavone (Nobiletin) ^a^	[36,42]	0.74
**83**	51.847	433.1485		403.1021, 388.0773	432.1413	432.1420	C_22_H_24_O_9_	3′,4′,3,5,6,7,8-Heptamethox-yflavone	[36]	1.79
**84**	52.030	343.1174		328.0927, 285.0749	342.1101	342.1103	C_19_H_18_O_6_	5,6,8,4′-Tetramethoxyflavone	[54]	0.63
**85**	52.381	343.1175		328.0919, 313.0705	342.1102	342.1103	C_19_H_18_O_6_	4′,5,7,8-Tetramethoxyflavone	[54]	0.36
**86**	53.099	373.1281		358.1007, 343.1182	372.1208	372.1209	C_20_H_20_O_7_	5,6,7,8,4′-Pentamethoxyflavone, (Tangeretin) ^a^	[36,42]	0.25
**Common Compounds**										
**87**	1.711	116.0705			115.0632	115.0633	C_5_H_9_NO_2_	Proline	[38]	0.83
**88**	2.849	118.0865			117.0792	117.0790	C_5_H_11_NO_2_	Valine	[38]	−1.83
**89**	4.328	132.1016			131.0943	131.0946	C_6_H_13_NO_2_	Isoleucine	[38]	2.32
**90**	4.678	132.1019			131.0947	131.0946	C_6_H_13_NO_2_	Leucine	[38]	−0.32
**91**	5.813	182.0810			181.0737	181.0739	C_9_H_11_NO_3_	tyrosine	[38]	0.83
**92**	9.561	166.0859			165.0786	165.0790	C_9_H_11_NO_2_	Phenylalanine	[38]	2.36
**93**	17.316	205.0969			204.0896	204.0899	C_11_H_12_N_2_O_2_	Tryptophan	[38]	1.34
**94**	25.552	113.0597			112.0524	112.0524	C_6_H_8_O_2_	Sorbic acid ^a^	/	0.25

^a^ Compound identified with standards; ^b^ The error (ppm < 5 ppm) was obtained via the accurate mass data and formula predictor software of TOF mass spectrometer.MW (Mea.) = Molecular weight (measured); MW (MFG) = Molecular weight (molecular formula generated).

**Table 2:** Binding affinity (%) and docking score (kj/mol) of the ligands in Simo decoction (SMD) with human serum albumin (HSA).

No.	Ligand	Binding Affinity	Docking Score	
			Site I	Site II
A	Norisoboldine	26.1	−34.7	−36.1
B	Eriocitrin	14.2	−39.7	−30.5
C	Neoeriocitrin	15.3	−38.9	−31.8
D	Narirutin	15.5	−40.6	−31.4
E	Hesperidin	11.6	−39.3	−30.1
F	Naringin	13.9	−39.7	−33.9
G	Neohesperidin	12.8	−39.3	−30.1
H	Hesperitin-7-*O*-glucoside	9.8	−36.4	−35.5
I	Linderane	22.5	−34.3	−36.0
J	Poncirin	16.7	−38.9	−31.0
K	Costunolide	19.6	−33.5	−35.9
L	Nobiletin	14.7	−32.2	−33.5
M	Tangeretin	12.9	−31.4	−34.7
Drugs ^a^	Warfarin	−	−33.5	−
	Ibuprofen	−	−	−32.2

^a^ The drugs warfarin and ibuprofen were specific ligands for site I and site II, respectively.
